# Efficacy and tolerability of adding coenzyme A 400 U/d capsule to stable statin therapy for the treatment of patients with mixed dyslipidemia: an 8-week, multicenter, double-Blind, randomized, placebo-controlled study

**DOI:** 10.1186/1476-511X-13-1

**Published:** 2014-01-02

**Authors:** Jiangtao Lai, Bifeng Wu, Tianming Xuan, Zhong Liu, Junzhu Chen

**Affiliations:** 1 Department of Cardiology, First Affiliated Hospital, College of Medicine, Zhejiang University, 79# Qingchun Road, 310003 Hangzhou, China

**Keywords:** Coenzyme A, Hypertriglyceridemia, Dyslipidemia, Statin, Lipoprotein, Combination therapy

## Abstract

**Background:**

Patients with mixed hyperlipidemia usually are in need of combination therapy to achieve low-density lipoprotein cholesterol (LDL-C) and triglyceride (TG) target values for reduction of cardiovascular risk. This study investigated the efficacy and safety of adding a new hypolipidemic agent, coenzyme A (CoA) to stable statin therapy in patients with mixed hyperlipidemia.

**Methods:**

In this multi-center, 8-week, double-blind study, adults who had received ≥8 weeks of stable statin therapy and had hypertriglyceridemia (TG level at 2.3-6.5 mmol/L) were randomized to receive CoA 400 U/d or placebo plus stable dosage of statin. Efficacy was assessed by the changes in the levels and patterns of lipoproteins. Tolerability was assessed by the incidence and severity of adverse events (AEs).

**Results:**

A total of 304 patients with mixed hyperlipidemia were randomized to receive CoA 400 U/d plus statin or placebo plus statin (n = 152, each group). After treatment for 8 weeks, the mean percent change in TG was significantly greater with CoA plus statin compared with placebo plus statin (-25.9% vs -4.9%, respectively; *p* = 0.0003). CoA plus statin was associated with significant reductions in TC (-9.1% vs -3.1%; *p* = 0.0033), LDL-C (-9.9% vs 0.1%; *p* = 0.003), and non- high-density lipoprotein cholesterol (-13.5% vs -5.7%; *p* = 0.0039). There was no significant difference in the frequency of AEs between groups. No serious AEs were considered treatment related.

**Conclusions:**

In these adult patients with persistent hypertriglyceridemia, CoA plus statin therapy improved TG and other lipoprotein parameters to a greater extent than statin alone and has no obviously adverse effect.

**Trial registration:**

Current Controlled Trials ClinicalTrials.gov ID NCT01928342.

## Introduction

Hyperlipidemia plays important roles in the development and progression of atherosclerosis and coronary artery disease (CAD) [1,2]. Modulating lipid levels mostly focused on the control of low-density lipoprotein cholesterol (LDL-C) with 3-hydroxy-3-methylglutaryl coenzyme A reductase inhibitors (statins) has been shown to be clearly efficacious in the treatment and prevention of CAD [3]. However, despite increasing use of statins, even in optimal doses to achieve target LDL-C reduction, considerable residual risk remains. Such risk resides in elevated levels of triglycerides (TG), and subnormal levels of atheroprotective high-density lipoprotein cholesterol (HDL-C), and treatment combining a statin with another lipid-modifying agent may be required for mixed hyperlipidemia [4-7].

However, combination therapies, including fibrates, niacin, or omega-3 fatty acids (OM3-FAs) in addition to statin treatment, are often recommended but less often used due to concerns with cost, tolerability and compliance [8]. Impairment of liver function, increase in creatine phosphokinase (CPK) levels, and more serious complications such as myositis and rhabdomyolysis are more frequent when fibrates are combining used with a statin [9-11]. Many patients have difficulty tolerating niacin because of relatively benign but troublesome adverse events, namely, flushing, and niacin may exacerbate hyperuricemia, glucose intolerance, and hepatic dysfunction when combining with a statin [12-14]. OM3-FAs have been shown their well tolerability as an adjunct to statin therapy in numerous studies [15-17]. However, it was also reported that OM3-FAs can produce an increase in LDL in some patients [17-20]. So, there is ongoing research into new, safer and more effective agents to be used alone or in combination with existing cardiovascular drugs [21].

Coenzyme A (CoA) functions as an acyl group carrier and assists in transferring fatty acids from the cytoplasm to mitochondria [22]. It is also involved in the oxidation and catabolism of fatty acids [23,24]. Previous researches revealed that insufficiency of CoA in vivo influenced fatty acid β-oxidation catabolism and impaired clearance of TG from plasma [23,24], which was supposed to be one plausible reason resulting in type IIb and type IV hyperlipoproteinemia. Moreover, studies on animals have given evidence to prove that supplement of CoA had normalizing activity on plasma lipids in dyslipidemia [25,26]. In a previous multicenter study we conducted in 2008, it was found that oral CoA 400 U/d monotherapy effectively lowered serum TG levels in hypertriglyceridemia patients without increasing adverse effects when compared with placebo [27]. So far there has no sufficient clinical research data to support the efficacy and saftey of oral CoA when combining used with statin in mixed dyslipidemia patients. The present study assessed the efficacy and tolerability of CoA capsule in combination with one stable statin therapy for lowering TG and other lipid and lipoprotein levels in patients with mixed dyslipidemia.

## Methods

### Patients

Eligible patients were male or female between 18 to 80 years of age who had been receiving a normal dose of a statin (atorvastatin 20 mg/d, rosuvastatin 10 mg/d, simvastatin 40 mg/d, pravastatin 40 mg/d, pitavastatin 4 mg/d, fluvastatin 80 mg/d, or lovastatin 40 mg/d) for the control of LDL-C levels for at least 8 weeks before screening and had a fasting TG level at 2.3-7.0 mmol/L. Main exclusion criteria included hepatic dysfunction with elevations of alanine aminotransferase (SGPT) or aspartate aminotransferase (SGOT) >2 times the upper limit of normal (ULN); renal impairment as defined by serum creatinine ≥179 μmol/l; unexplained serum creatine phosphokinase (CPK) >2 times ULN; poorly controlled hypertension (resting systolic blood pressure ≥ 180 mmHg and/or diastolic blood pressure ≥ 110 mmHg at 2 consecutive visits); pregnancy; breast-feeding; women of childbearing potential not using chemical or mechanical contraception; history of alcohol or drug dependence; history of an acute coronary syndrome within 6 month of screening; uncontrolled diabetes; hypothyroidism; New York Heart Association class IIIb or IV heart failure or with a left ventricular ejection fraction known to be <30%; history of receiving therapies with other non-statin lipid-modifying drugs (e.g. fibrates, niacin, or fish oils) within 2 months; or history of adverse events associated with test agents.

### Study design

This phase 3, randomized, double-blind, placebo-controlled, multicenter study was conducted between April 2012 and July 2013 in 10 centers in China. The study consisted of 5 clinic visits: 1 screening visit, 2 visit during the lead-in/baseling period, and 2 visits during double-blind treatment. The study protocol and consent form were reviewed and approved by the appropriate institutional ethics committee at each study site. The study was conducted in accordance with the Good Clinical Practice Guidelines, and the Declaration of Helsinki. Prior to any study procedures, written informed consent was obtained from each study subject by investigators of each center. The trial has been registered under Clinical-Trials.gov Identifier NCT01928342 (http://clinicaltrials.gov/show/NCT01928342).

At screening (week -4), patients meeting the initial eligibility criteria received the same open-label statin treatment as before with the dosage remaining stable, which was continued for the remainder of the study. In addition, they received dietary counseling on the NCEP Therapeutic Lifestyle Changes diet [28].

After the lead-in phase, patients whose compliance (measured by the number of capsules consumed relative to the number expected to be consumed) with statin therapy was ≥80% and who had TG level at 2.3-7.0 mmol/L with changes in TG levels between weeks -4 and -1 less than 20% were randomized to receive 8 weeks of double-blind CoA capsule 400 U/d or placebo (Meiyou Pharmaceutical Group Co., Ltd., Shanghai, China). Patients were allocated numbers as they entered the study, and a computer-generated randomization scheme was used to determine treatment allocation as each patient became eligible. Treatment codes for each patient were provided to each center in a sealed envelope, and investigators were unaware of the treatment codes until the envelope was opened at the randomization visit.

Follow-up visits occurred at 4 and 8 weeks after initiation of therapy. A routine physical examination including height, weight, heart rate, and blood pressure was performed at each visit. Meanwhile, fasting (at least 12 hours) blood samples were collected. Medication compliance and the occurrence of adverse events (AEs) were assessed. Compliance was assessed using a capsule count at each visit. The causal relationship of adverse reactions to the treatment was assessed by the investigators. Education on healthy lifestyles was reinforced.

### Outcome measures and safety assessments

The primary endpoint was the percentage change of serum TG level from the baseline to the end of treatment. The secondary endpoint included the percentage changes from the baseline to the end of treatment in serum TC, LDL-C, HDL-C, and non-HDL-C levels.

Fasting blood samples were collected at weeks -4, -1, 4, and 8 for lipid profile and for clinical chemistry (including SGPT, SGOT, CPK, and serum creatinine) analyses. Central laboratory was used in the study. All blood samples were sent to the central laboratory in insulated packaging within 48 hours. Serum lipids were analyzed on a Hitachi 7600–210 analyzer (Hitachi High-Technologies, Tokyo, Japan) by a specialist who was unaware of this study. Other laboratory assessments included hematology (Sysmex XZ-2100 Hematology Analyzer, Sysmex Medica Co., Ltd., Hyogo, Japan); chemistry; urinalysis (Aution Max AX-4280 automated urine test-strip analyzer, ARKRAY, Inc., Kyoto, Japan); pregnancy testing for women of childbearing potential (chemiluminometric immunoassay). Quality control procedures were applied in line with the calibration and control requirements specified for each assessment and executed according to Good Laboratory Practice.

Tolerability assessments were based on reported AEs, physical examinations, and clinical laboratory evaluations such as liver enzyme elevation >3 × ULN or CPK elevation >10 × ULN. The relationship of AEs to the treatment was assessed (categorized as not related, unlikely, possibly, probably, or definitely related to study drug). Compliance was assessed using a capsule count at each visit.

### Statistical analyses

On the basis of the results of a phase 2 study [27], an evaluable sample of 286 (143 per treatment group) was expected to provide >80% power (2-sided α = 0.05) to detect a 10% difference in the mean percent change in TG levels between the 2 treatment groups (assumed pooled SD, 30%). To account for patient attrition and noncompliance of 5%, ≥302 patients were expected to be randomized.

The intent-to-treat (ITT) population included all randomized patients. Efficacy analyses involved all patients in the ITT population who received at least 1 dose of study medication and provided at least 1 post-randomization blood sample. The last-observation-carried-forward (LOCF) method was used to impute missing nonbaseline data for patients who did not complete the treatment period. Percent changes from baseline were evaluated by analysis of variance (ANOVA) with factors fitted for treatment and the pre-dose (baseline) lipid parameter value as a covariate. The results were presented as least squares means (LSM) and the difference between the LSM. Data were analyzed in SAS/STAT, version 9.13.

All patients who received at least 1 dose of double-blind study drug and returned to the clinic for at least 1 safety assessment after randomization were included in the safety population. The Fisher exact test (2-tailed) was used to compare the incidence of AEs between treatment groups.

## Results

### Patient characteristics

**Figure 1:**
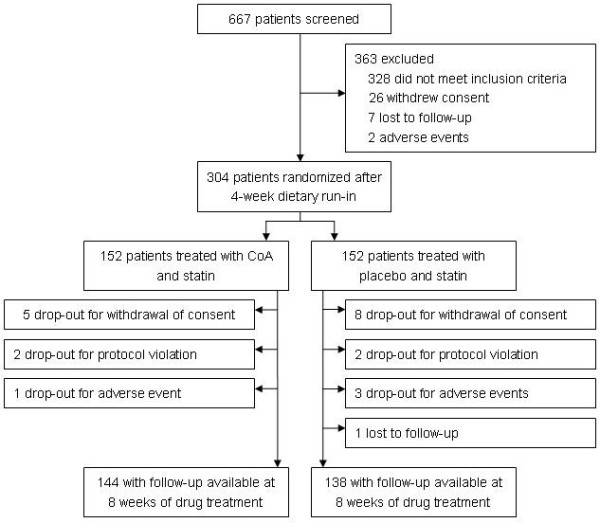
Patient disposition throughout the study.

Of 667 patients screened, 304 were randomized into the treatment groups (152 to CoA plus statin, 152 to placebo plus statin). Three hundred and sixty-three patients were discontinued before randomization: 328 subjects did not meet inclusion criteria, 26 withdrew their consents, 7 were lost to follow-up, and 2 dropped out for adverse events. The efficacy-evaluable and safety populations included 152 patients in each study group, who received at least 1 dose of the study drug and returned for at least 1 evaluation after randomization (ITT population). Patient disposition is summarized in Figure 1.

The number of patients completing the study was comparable in the CoA (144/152 [94.7%]) and placebo (138/152 [90.8%]) groups. There were 8 non-completes in the CoA group: 1 patient discontinued due to AE, 5 withdrew consent, and 2 was withdrawn for protocol violation. There were 14 non-completes in the placebo group: 3 patients discontinued due to AEs, 8 withdrew consent, 2 was withdrawn for noncompliance with the protocol, and 1 was lost to follow-up. For the 22 patients who withdrew during the double-blind treatment period after providing at least 1 postrandomization blood sample for lipid analysis, the LOCF method was used to impute values for the missing data points.

The efficacy-evaluable and safety populations had a mean age of 54.0 years and were 55.3% (168/304) male. Demographics and baseline characteristics of patients in the study population were demonstrated in Table 1. The 2 groups were comparable in age, sex, body mass index, blood pressure, risk factors, and medications, including the types of statins used. With the exception of the TC, which was significantly higher in the CoA group compared with the placebo group (*P* = 0.034), there were no significant differences in any lipid/lipoprotein level between treatment groups at baseline in the efficacy-evaluable population.

**Table 1:** Demographics and baseline characteristics of patients in the study population

**Characteristic**	**Statin + CoA (n = 152)**	**Statin + Placebo (n = 152)**
Age, years	54.8 ± 12.1	53.1 ± 13.1
Sex, n (%)		
Male	81 (53.3)	87 (57.2)
Female	71 (46.7)	65 (42.8)
BMI, kg/m^2^		
Male	26.3 ± 2.8	25.5 ± 2.4
Female	24.7 ± 2.9	24.1 ± 2.8
SBP, mmHg	131.4 ± 12.4	128.5 ± 13.9
DBP, mmHg	80.4 ± 9.1	78.1 ± 8.9
Risk factors, n (%)		
Type 2 DM	23 (15.1)	28 (18.4)
Hypertension	62 (40.8)	49 (32.2)
Coronary artery disease	18 (11.8)	15 (9.9)
Stroke	3 (2.0)	0 (0)
Medications, n (%)		
ACEIs/ARBs	38 (25.0)	27 (17.8)
β-blockers	32 (21.1)	20 (13.2)
Calcium channel blockers	40 (26.3)	24 (15.8)
Diuretics	7 (4.6)	1 (0.7)
Insulin	5 (3.3)	4 (2.6)
Oral hypoglycemic agents	28 (18.4)	26 (17.1)
Platelet aggregation inhibitors	25 (16.4)	18 (11.8)
Organic nitrates	2 (1.3)	3 (2.0)
Statins		
Atorvastatin	96 (63.2)	94 (61.8)
Rosuvastatin	12 (7.9)	15 (9.9)
Simvastatin	29 (19.1)	34 (22.4)
Fluvastatin	14 (9.2)	9 (5.9)
Lovastatin	1 (0.7)	0 (0.0)

All values are mean ± standard deviation (SD) unless otherwise specified.

NOTE: CoA = coenzyme A; BMI = body mass index; SBP = systolic blood pressure; DBP = diastolic blood pressure; DM = diabetes mellitus; ACEI = angiotensin-converting enzyme inhibitor; ARB = angiotensin receptor blocker.

### Efficacy analyses

**Figure 2:**
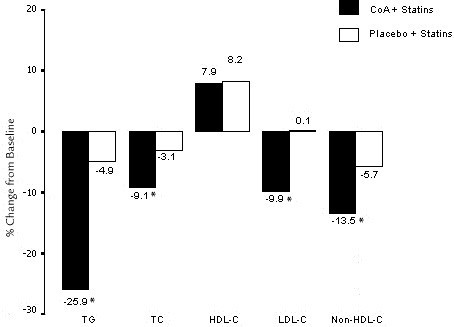
**Mean percent change in triglyceride (TG), total cholesterol (TC), high-density lipoprotein cholesterol (HDL-C), low-density lipoprotein cholesterol (LDL-C), and non-HDL-C from baseline to the end of treatment.** CoA = coenzyme A. *P < 0.01.

Changes in regard to lipid end points are presented in Table 2 and Figure 2. The percent change from baseline in TG, the primary outcome variable, was significantly better with CoA plus statin compared with placebo plus statin (-25.9% vs -4.9%, respectively; *p* = 0.0003). After treatment for 8 weeks, the mean percent change in TC levels was -9.1% in the CoA group, compared with -3.1% in the placebo group (*p* = 0.0033). The mean percent change in LDL-C levels was -9.9% in the CoA group and 0.1% in the placebo group in the end of study (*p* = 0.003). HDL-C changed by a mean of 7.9% in the CoA group, compared with a mean change of 8.2% in the placebo group (*p* = 0.94). CoA plus statin was associated with a mean percent change in non-HDL-C of -13.5% after 8-week treatment, compared with -5.7% in the group receiving statin plus placebo (*p* = 0.0039).

**Table 2:** Percent change from baseline to study visits in efficacy parameters

**Variable**		**Statin + CoA (n = 152)**	**Mean percent change, % ± SE (median)**	**Statin + Placebo (n = 152)**	**Mean percent change, % ± SE (median)**	***p *** **value**
TG, mmol/L	Baseline	3.58 ± 1.26		3.42 ± 1.12		
	Week 4	2.71 ± 1.70	-24.70 ± 37.69 (-32.69, range -228.83 ~ 90.91)	3.07 ± 1.93	-9.77 ± 48.32 (-16.59, range -245.79 ~ 81.82)	0.0029
	Week 8	2.69 ± 2.21	-25.86 ± 50.35 (-36.81, range -386.65 ~ 85.59)	3.23 ± 1.99	-4.87 ± 48.77 (-9.63, range -234.51 ~ 84.49)	0.0003
TC, mmol/L	Baseline	5.37 ± 1.17*		5.08 ± 1.22		
	Week 4	4.84 ± 1.10	-8.22 ± 18.62 (-8.67)	4.78 ± 1.21	-4.66 ± 17.16 (-4.12)	0.085
	Week 8	4.79 ± 1.07	-9.13 ± 18.95 (-9.43)	4.86 ± 1.25	-3.13 ± 16.16 (-3.21)	0.0033
HDL-C, mmol/L	Baseline	1.20 ± 0.31		1.15 ± 0.30		
	Week 4	1.24 ± 0.33	6.17 ± 25.87 (1.59)	1.19 ± 0.32	4.86 ± 20.41 (0.00)	0.63
	Week 8	1.26 ± 0.36	7.92 ± 28.27 (1.84)	1.23 ± 0.38	8.16 ± 25.87 (1.91)	0.94
LDL-C, mmol/L	Baseline	2.91 ± 0.98		2.71 ± 0.92		
	Week 4	2.61 ± 0.94	-6.35 ± 30.90 (-10.85)	2.57 ± 0.94	-2.51 ± 26.46 (-3.56)	0.25
	Week 8	2.53 ± 0.92	-9.90 ± 29.73 (-12.21)	2.63 ± 0.90	0.10 ± 28.28 (-1.46)	0.003
non-HDL-C, mmol/L	Baseline	4.18 ± 1.10		3.92 ± 1.16		
	Week 4	3.60 ± 1.08	-11.64 ± 23.43 (-13.79)	3.59 ± 1.17	-6.61 ± 22.97 (-6.41)	0.06
	Week 8	3.53 ± 1.06	-13.45 ± 24.02 (-15.39)	3.64 ± 1.21	-5.73 ± 22.14 (-5.28)	0.0039

All values are mean ± standard deviation (SD) unless otherwise specified.

Note: CoA = coenzyme A; TG = triglyceride; TC = total cholesterol; HDL-C = high-density lipoprotein cholesterol; LDL-C = low-density lipoprotein cholesterol. *p* values indicate the percent change between the two groups. *p = 0.034, between-group comparison of baseline TC level.

### Safety analyses

There was no significant difference between groups in the proportion of patients experiencing AEs (Table 3). Serious AE (SAE) occurred in 1/152 (0.7%) patient in the placebo group and no SAE in the CoA group. The only SAE in the placebo group was a 61-year-old woman hospitalized for an exacerbation of chest pain and was not considered by the investigator to be related to study treatment. The proportion of patients who discontinued study treatment because of AEs did not differ statistically – in the CoA plus statin group 0.0%, in the placebo plus statin group 2.6% (Table 3). No AEs involved myopathy (CPK >10 × ULN) or rhabdomyolysis. The combination of CoA and statin had no significant effect on creatinine level during the course of the trial.

**Table 3:** Incidence of adverse events

**Variable**	**Total (n = 304)**	**Statin + CoA (n = 152)**	**Statin + Placebo (n = 152)**
Any AE	25	14	11
Serous AEs	1	0	1
Patients who experienced any AE	23	12	11
Lead to treatment discontinuation	4	0	4
Most common AEs			
Abdominal distention	1	1	0
Nausea	2	1	1
Chest pain	1	0	1
Elevated liver enzymes	1	1	0
Elevated creatine phosphokinase	7	3	4
Upper respiratory tract infection	5	4	1

Note: CoA = coenzyme A; AE = adverse event.

There were no cases of clinically significant increases in hepatic transaminase levels (>3 × ULN) in either group. There was a numerically higher incidence of mildly elevated SGPT in the group that received CoA plus statin compared with the group that received statin only (0.7% [1/152] vs 0.0% [0/152], respectively; *p* = NS). The incidence of mildly elevated CPK was also comparable between the 2 groups (2.0% [3/152] vs 2.6% [4/152], respectively; *p* = NS). The group mean changes from baseline in SGPT, SPOT, creatinine, and CPK were small and did not differ between the 2 groups (data not shown).

## Discussion

Hyperlipidemia is an important contributory factor to development of atherosclerosis, and as such is recognized as a major risk factor for CAD. Consequently, lipid lowering therapy is very important for the prevention and treatment of CAD [1,2]. Even though statins are recommended as first-line drug treatment for patients with high risk of CAD [29,30], recent studies reported the importance of lowering serum TG independent of other lipid variables [6,31,32]. Results from clinical intervention studies showed that triglyceride lowering therapies were associated with reduction in non-fatal MI, revascularization, and all-cause mortality [6]. In 2002, NCEP ATP III guidelines have identified non-HDL-C as a secondary treatment target for CAD risk reduction in individuals with significant evlevations in plasma TG levels (≥2.26 mmol/L), with a target 0.78 mmol/L higher than the LDL-C goal [28]. However, hypertriglyceriemia or non-HDL-C level may not be adequately controlled with statin monotherapy. Concerning with safety and tolerability of TG-lowering agents being used nowadays, especially in combination therapies, many patients with mixed hyperlipidemia did not achieved combined LDL-C and non-HDL-C goals [33]. So, safer and better tolerable agents are being expected to target the residual risk of hypertriglyceridemia, high non-HDL-C, or low HDL-C level [24]. The present study investigated the efficacy and tolerability of coadministration of a new hypolipidemic agent, CoA with one statin for lowering TG and other lipoproteins in patients with persistent hypertriglyceridemia despite statin therapy.

In a previous placebo-controlled trial in patients with moderate hypertriglyceridemia, administration of CoA for 8 weeks resulted in 36.1% reduction in TG compared with placebo (*p* < 0.0001), without any obviously adverse effect [27]. In the present study, coadministration of CoA with statin in patients with hypertriglyceridemia despite statin therapy was associated with a significantly greater reductions in TG compared with statin alone (*p* < 0.001). CoA was also associated with a significant reduction in TC, LDL-C and non-HDL-C (all, *p* < 0.01). Considering the combined effects of reducing TG and atherogenic lipoproteins, the addition of CoA to statin therapy may provide additional clinical benefits in patients with mixed dylipidemia [4-6,31,34,35].

**Figure 3:**
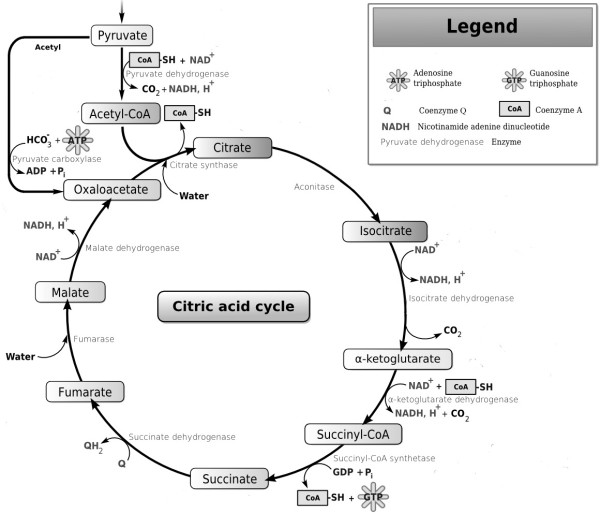
The role of coenzyme A in tricarboxylic acid cycle (citric acid cycle).

The mechanism of lipid modifying effects with CoA has not been elucidated thoroughly. CoA is one of the most important biologically active compounds which are essential for metabolism of the three major forms of energy (fat, carbohydrates, and protein). It presents in all living cells that functions as an acyl group carrier and plays a central role in the tricarboxylic acid cycle (Figure 3). CoA has a clearly defined role in promoting fat decomposition, regulating the synthesis of key enzyme of fatty acids synthetic metabolism, as well as in facilitating fatty acid β- oxidation. As a cofactor for a number of oxidative and biosynthetic reactions in intermediary metabolism, CoA is also involved in the oxidation of fatty acids. Therefore, it is presume that CoA functions as regulator on the lipid metabolism, and has a lipid lowering effect in hyperlipidemia [36].

Previous studies showed that intravenous administration of CoA could accelerate TG catabolism and thereby lower TG level by inducing fatty acid oxidation response. It was suggested that CoA injection could effectively lower postprandial serum triglyceride level increasing in patients with fasting hypertriglyceridemia [23,24]. Although sufficient evidences supported the lipid lowering effect of CoA, it was inconvenient for patients to receive chronic intravenous CoA administration. So, oral CoA capsule was developed. Animal studies showed that fasting TC and LDL-C levels were significantly reduced with oral CoA administration at 800 and 1500 U/kg per day for 20 days, and TG level was also decreased at a higher dose, suggesting its potential therapeutic role in dyslipidemia [25,26]. In the previous study, oral CoA 400 U/d effectively lowered serum TG levels in hypertriglyceridemia patients [27]. The present study confirms the lipid lowering effect of CoA as reported previously and demonstrated that the combination of CoA and statin improved the overall lipid profile without attenuating the efficacy of the statin.

In this study, both treatments were well tolerated. The incidence and nature of AEs were similar in the 2 treatment groups. The safety profile of CoA administered with statin was consistent with the known safety profile of CoA administered alone [27]. In the statin plus CoA group, the most frequently occurring AEs were upper respiratory tract infection and elevated CPK, none of which differed significantly from the placebo group. Considering such AEs were not reported in the previous study with CoA, and the combining use of statin in this study, the relationship of elevated CPK or upper respiratory tract infection to CoA use cannot be finally determined yet.

### Study limitations

This is the first study to evaluate the efficacy and clinical safety of CoA capsule combining with statin in subjects with mixed dyslipidemia. The current study has a few limitations. The first limitation of this study was the short duration of double-blind treatment (8 weeks). Some future studies with longer follow-up duration may better characterize the long-term efficacy and tolerability of coadministration of CoA and statin, and the impact of such coadministration on clinical outcomes. Secondly, the patients enrolled in this trial are all Chinese subjects, so the results of this trial may not be directly extrapolated to other races without any international multicenter clinical trial result. Finally, although a possible explanation for the results has been suggested, the underlying mechanism of the findings cannot be completely clarified by the present data. However, the elucidation of the exact mechanism of the difference is beyond the purpose of this study and will hopefully be evaluated by a further investigation.

## Conclusions

In the present study, the addition of CoA 400 U/d to ongoing normal dose of statin was effective in providing additional lowering of TG, TC, LDL-C, and non-HDL-C levels in patients with mixed hyperlipidemia. The combination of CoA and statin was well tolerated.

## Abbreviations

TG: Triglyceride; CoA: Coenzyme A; OM3-FAs: Omega-3 fatty acids; AEs: Adverse events; LDL-C: Low-density lipoprotein-cholesterol; HDL-C: High-density lipoprotein cholesterol; TC: Total cholesterol; SGPT: Serum alanine aminotransferase; SGOT: Serum aspartate aminotransferase; ULN: Upper limit of normal; CPK: Creatine phosphokinase; ITT: Intention to treat; LOCF: Last-observation-carried-forward; LSM: Least squares means.

## Competing interests

The authors declare no conflict of interest. This study was supported financially by Institute for Herb Biomedical Engineering in Shanghai and Public Technology Research and Social Development Project of Zhejiang Province (No. 2013C33118).

## Authors’ contributions

JL and ZL conducted the clinical part of the study, JL, TX and JC designed the study, JL, TX and BW analyzed the data and wrote the manuscript. All authors read and approved the final manuscript.
